# Review on data analysis methods for mesoscale neural imaging *in vivo*

**DOI:** 10.1117/1.NPh.9.4.041407

**Published:** 2022-04-15

**Authors:** Yeyi Cai, Jiamin Wu, Qionghai Dai

**Affiliations:** Tsinghua University, Department of Automation, Beijing, China

**Keywords:** data analysis, mesoscale neural imaging, pipeline

## Abstract

**Significance:**

Mesoscale neural imaging *in vivo* has gained extreme popularity in neuroscience for its capacity of recording large-scale neurons in action. Optical imaging with single-cell resolution and millimeter-level field of view *in vivo* has been providing an accumulated database of neuron-behavior correspondence. Meanwhile, optical detection of neuron signals is easily contaminated by noises, background, crosstalk, and motion artifacts, while neural-level signal processing and network-level coordinate are extremely complicated, leading to laborious and challenging signal processing demands. The existing data analysis procedure remains unstandardized, which could be daunting to neophytes or neuroscientists without computational background.

**Aim:**

We hope to provide a general data analysis pipeline of mesoscale neural imaging shared between imaging modalities and systems.

**Approach:**

We divide the pipeline into two main stages. The first stage focuses on extracting high-fidelity neural responses at single-cell level from raw images, including motion registration, image denoising, neuron segmentation, and signal extraction. The second stage focuses on data mining, including neural functional mapping, clustering, and brain-wide network deduction.

**Results:**

Here, we introduce the general pipeline of processing the mesoscale neural images. We explain the principles of these procedures and compare different approaches and their application scopes with detailed discussions about the shortcomings and remaining challenges.

**Conclusions:**

There are great challenges and opportunities brought by the large-scale mesoscale data, such as the balance between fidelity and efficiency, increasing computational load, and neural network interpretability. We believe that global circuits on single-neuron level will be more extensively explored in the future.

## 1 Introduction

Recording neural activities *in vivo* with optical systems and genetically encoded fluorescence indicators provides an observation window for neural scientists to understand the signal processing procedure of individual neurons and the circuitry of neural network in action. Compared with electrophysiological methods, optical imaging *in vivo* is typically less invasive and could record several brain areas up to millimeter-level field of view (FOV) at cellular resolution.^1^^,^^2^ Animal surgeries, such as cranial window, thinned skull, or crystal skull^3^^,^^4^ provide the optical imaging window for one-photon imaging to achieve single-cell resolution in the superficial cortex, such as layer 2/3. Optical neural imaging has thus been used to investigate neural structure change,^5^^,^^6^ brain state alteration,^7^ and information flow while the animal performs a specific task. Profound discovery has been made by *in vivo* neural imaging on crucial neuroscience issues, including perceptual input processing,^8^^,^^9^ motion,^10^^,^^11^ learning,^12^^,^^13^ memory,^14^^,^^15^ and decision making.^16^

To date, multiple imaging modalities are capable of mesoscale recording with single-cell resolution and millimeter-level FOV, such as single-photon widefield microscopy,^3^^,^^17^ multi-photon microscopy,^18^^,^^19^ light-field microscopy,^20^^,^^21^ and light-sheet microscopy.^22^ A recent review^2^ has described the mesoscale imaging techniques and related animal models in detail, which also points out its increasing importance in neuroscience. While the challenges and opportunities brought by the large-scale mesoscale data have been gradually realized by the community,^2^ there is still not a comprehensive review about the existing mesoscale analysis methods, remaining problems, and potential future directions. Here, we intended to provide a general data analysis pipeline for cellular level mesoscale neural imaging without focusing on any specific imaging modality. We emphasize the commonalities between different image systems. For instance, the data analysis always starts with neural images from detectors and ends by useful information extracted from calcium signals. Some imaging modalities have their unique preprocess algorithm before acquiring the neural image, such as slice stitching in light-sheet microscopy and volume reconstruction in light-field microscopy. These are beyond the scope of this work. However, we do illustrate the specific priors in data analysis processing, which can be considered based on different imaging modalities, during our detailed descriptions of each processing step. For readers who might be not familiar with microscopy systems, we briefly review the image modalities used for *in vivo* neural dynamic imaging in the following paragraphs.

In the past decades, benefitting from the rapid development of both microscopic systems^17^^,^^23^^–^^25^ and fluorescent indicators,^26^^,^^27^ *in vivo* neural imaging has been extending its capability in faster sampling speed, higher resolution, larger FOV, and lower phototoxicity.^28^ The simple wide-field microscopy could cover several adjacent cortex areas,^29^ but it does not typically achieve cellular resolution because of scattering and aberration. Several recent works have shown capabilities to extract single-cell information in wide-field data even with strong background fluorescence by matrix factorization and deep learning.^30^^,^^31^ In addition, animal models and new fluorescence indicators with specific labeling strategies can greatly reduce the background fluorescence in normal wide-field microscopes, facilitating single-cell resolution neural recoding with simple systems, e.g., layer-specific labeling,^3^ and soma-targeted sensors.^32^^,^^33^ In addition, with special optical designs, several works have been done to further increase the resolution or depth of field with better fidelity to retrieve the single-cell neural traces. For instance, in the RUSH system,^17^ a 5×7 camera array was tiled to cover centimeter-level FOV and reach 0.8-μm resolution with dense sampling density and layer-specific neuron labeling; the COSMOS macroscope^4^ uses multifocal optical sampling to record in-focus projection of 1  cm×1  cm×1.3  mm volume at near cellular resolution (1–15 neurons/unit). To examine the on-focus slice only, confocal microscopy^34^ and light sheet^35^ microscopy were designed by either blocking out the out-of-focus light or illuminating a thin slice of the tissue from the side. These techniques have enabled optically sectioning of the brain tissue with three-dimensional (3D) resolving power through scanning strategy. Nevertheless, the resolution of single-photon microscopy degrades tremendously with the increase of penetration depth,^36^ thus it was only useful in detecting neurons in shallow cortex layers. Multiphoton microscopy (MPM), on the other hand, holds the advantage in penetration depth^37^ and low photodamage. Multiple photons with lower energy cooperate to excite the fluorophore with a nonlinear absorption rate to light intensity. Hence, MPM was widely used to image deep mouse brain.^38^^,^^39^ Gradient index microlenses further extended the imaging depth to even deeper brain nuclear by affecting the optical path of the exited fluorescence.^40^

There have been emerging computational techniques for high-throughput 3D volumetric imaging.^41^ In two-photon microscopy, 3D imaging is accomplished by quickly scanning the sample with single-dot or slice excitation which could be either sequentially or randomly.^42^ Yet the sampling frequency is essentially limited by the control frequency of the mechanical actuator and the inertia of the optical system. An alternative approach was through multiplexing. A multi-focus microscope acquires multiple depth information by multi-focusing optical path^19^^,^^43^^–^^45^ or point-spread function engineering.^18^^,^^46^ Light-field microscopy captures the 3D information efficiently in a tomographic manner with extended depth of field along different angles.^47^^–^^49^ While scanning light-field microscopy significantly increases the spatial resolution in multi-cellular organisms,^20^ confocal light-field microscopy,^21^ or computational optical sectioning^50^ further increase its signal-to-background ratio in brain tissue. These techniques have enabled parallel volumetric imaging, capturing over thousands of neurons at cellular level with dozens of volumes per second.

Apart from benchtop microscopy systems where animals are head-fixed, head-mounted miniature microscopies would allow animals to freely move in experimental environments. Miniature microscopes could facilitate studies that are better performed in unrestrained subjects, such as spatial navigation, social behavior, and reward-seeking. To minimize the weight and size of the system, light-emitting diode, image acquisition chips, and miniatured lenses are commonly used in miniature microscopy. Progress has been made in miniature systems with millimeter-level FOV and near cellular resolution.^51^^,^^52^

Unlike electrophysiology detection, optical detection relies on the photon transmission of the genetically encoded indicators and the optical sensor. The optics and electrics conversion occurred twice during the imaging process, once by optical bio-indicator, once by the camera sensor. The indirectness of the signal detection results in potential signal corruption and reconstruction indispensability. In the meantime, mesoscale neural imaging usually features with an extremely large data throughput across multiple scales. These barriers of signal recovery have strengthened the essentiality of efficient and accurate computational approaches to extract high-fidelity neural activities from large-scale raw images captured by mesoscale imaging systems.

**Fig. 1:**
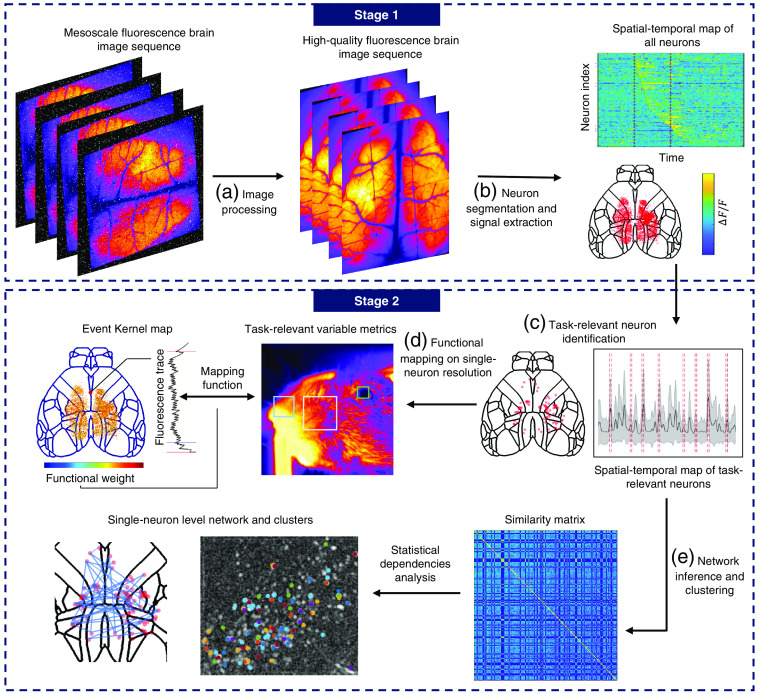
General pipeline of analyzing mesoscale fluorescence functional neural images. The whole pipeline is summarized into two main stages. The first stage targeted at spatial-temporal demixing of neural signals and the second stage targeted at data mining. Each stage contains a sequence of processes, which is framed up with a dashed-line box. (a) Image preprocesses includes three steps: motion registration, denoising, and hemodynamic correction. (b) After calcium signal extraction, the raw video sequence is decomposed into the spatial information of each neuron and their temporal fluorescence signals. (c) Task-relevant neurons only make up a small proportion of total neurons, thus should be recognized through statistical tests. (d) Linear and non-linear regressions map the test-relevant variables such as performance accuracy, gesture and choice into single-neuron traces, thus revealing the functional role of each neuron. (e) Similarity matrix based on correlation, cross entropy and causality could be used to analyze statistical dependencies between neurons and induce the neuron cluster community and neural network.

Here, we review recent data analysis methods for mesoscale intravital neural imaging along a general data-processing pipeline divided into two main stages (Fig. 1). Stage 1 includes several image processing procedures and outputs the compressed spatial-temporal single-neuron traces.^53^^,^^54^ Stage 2 includes various data-mining methods to interpret mesoscale neural signals both at the cellular level and network level. First, the video captured by the camera sensor should be registered to a template. Image sequences can be motion-blurred because of the heart-beating, breathing, or moving gestures of the animal. To identify specific neurons across a long term, all frames should be registered to a reference position. The second step is image denoising, the method of which depends on different signal-noise ratios (SNRs) and imaging modalities. Lower laser power is always preferred with *in vivo* experiments due to phototoxicity, in which case Poisson noise usually dominates over the readout noise and dark noise with high-speed high-sensitivity detectors. Under low-light conditions, computational denoising methods become indispensable because the noise can easily corrupt the down-stream analysis and interfere the interpretation of the neural activities. Calcium fluorescence signals might also be interfered by hemodynamic, which should be corrected before signal extraction. After this, the 4D/3D stack [3D/2D spatially and one-dimensional (1D) temporally] of the brain tissue is ready for neuron signal extraction. The goal of signal extraction is the demix of spatial-temporal information embedded in the fluorescence image. Neurons need to be spatially segmented from the brain tissue background, and their temporal traces are extracted from the temporal sequence of the image stack. These two steps could be performed sequentially by first determining the position or footprint of the neurons and averaging the relevant pixels for temporal traces, or parallelly by treating the spatial-temporal dimension as equivalent dimensions and utilizing the low-rank prior to perform a tensor-factorization. After the signal extraction step, the data size should be reduced to several megabytes (MBs) and could be represented as two 2D matrixes containing the temporal trace of neurons and their spatial footprints. The following analysis of the neuron trace could be diverse depending on the research problem. Generally, there are two levels of analysis. The first level is to investigate the single-neuron property, such as their tuning curves and the post-stimulus time histogram. The reaction patterns of these neurons may be used to analyze their relationship to certain stimuli or behavior. The second level of analysis extends the scale to local or global circuits formed by single neurons. These kinds of studies aim to infer the mesoscale functional network connection between the neurons to reconstruct the calculating strategy the neural circuit uses to accomplish specific signal processing missions.

In the following sections, we will review recent data analysis methods in each step. And we classify and evaluate different approaches in algorithm feasibility, scope of application, and precisions. The first stage which aims at a precise and efficient extraction of neuron traces is introduced in Sec. 2; in Sec. 3, we introduce the second stage which explores the underlying neural property and functional circuits through these mesoscale imaging data. Finally, in Sec. 4 we discuss the prospects and remaining challenges for mesoscale neural signal analysis.

## 2 Image Processing and Signal Extraction

### 2.1 Image Preprocess: Motion Registration, Denoise, and Hemodynamic Correction

Calcium imaging is often accompanied by motion artifacts, even if the animal was head-fixed and anesthetized. In head-fixed experiments, the non-rigid warping of the brain tissue could be caused by heart-beating, breathing, or the shrinking of the tissue from exposure to the laser. Moreover, with freely moving animals, the motion artifact becomes even more severe, because the posture changes induce relative movement of the head and objective lens. In long-term observation experiments, which expand several hours or days, as well as experiments involving different animal subjects, the distortion between frames becomes relatively severer.

**Fig. 2:**
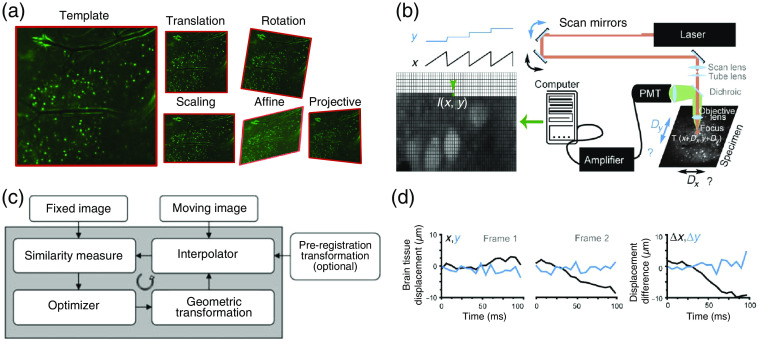
Inter-frame and intra-frame motion artifact correction. (a) Inter-frame motions are global transformations of the whole image which include rigid transformation (translation, rotation, and uniform scaling) and non-rigid transformation (scaling, affine, and projective). (b) Intra-frame motions render as pixel-wise displacement, which is usually caused by the motion during point scanning in multi-photon microscopies. (c) Diagram of intensity-based registration methodologies for inter-frame motion registration. (d) Example estimated trace produced by Lucas and Kanade algorithm, showing the displacement of the specimen in x (black) and y (cyan) directions in each frame and displacement difference between the two frames. Panels (b) and (d) are adapted from Ref. 56. Panel (c) is adapted from Ref. 55.

We usually assume the stability prior during the calcium extraction step. Thus, an efficient motion-correction algorithm improves the accuracy of neuron extraction. Motion registration is often conducted by transforming each frame toward a reference frame using a mapping function. Linear transformations could be described by a linear matrix. Consider a 2D images as an example (3D volume could be generalized through adding an element to the coordinate vector). The mapping function between the coordinates $x={\left[x_{1},x_{2},1\right]}^{T}$ and $x^{\prime }={\left[x_{1}^{\prime },x_{2}^{\prime },1\right]}^{T}$ of the corresponding sample points could be described as a 3×3 linear transformation matrix T, where $x^{\prime }=Tx$. The number of free parameters in T determines the transformation type, which could be divided into rigid transformation (translation and rotation) and non-rigid transformation (similarity, affine, and scaling)^55^ [Fig. 2(a)]. Nonrigid or nonlinear transformations could also be realized by first splitting the image into overlapping patches to perform rigid correction, and merging the patches inversely for an partition-based non-rigid transformation field.^57^ In head-fixed experiments, rigid transformations are usually sufficient for correction of a FOV smaller than 1 mm, and in mesoscale-imaging and freely moving animals, the non-rigid registrations are often demanded. 3D volume non-rigid registration raises the challenge of high computational load. The implementation of the graphic processing unit (GPU)-enhanced algorithms may be considered in large dataset registration missions. In some specific algorithms, the acceleration rate could be up to 100-fold.^58^

There are both inter-frame motion artifacts and intra-frame motion artifacts in neural imaging. The former exists widely in almost all kinds of microscopic modalities with the morphological shifting of the brain tissue. And the latter exists mainly in MPM because of the pixel-level temporal incoherence of sampling by point-scanning strategy [Fig. 2(b)]. Inter-frame motion artifacts are relatively more rigid than intra-frame motion artifacts, because there is usually no distortion within a single image, and a reference image is easier to find. A reference frame could be determined by visual inspection or averaging adjacent frames. All other frames could be registered to the reference frame.^59^ The features used for registration could be pixel intensity,^60^ extra structural channel,^61^ or exogenous landmark.^62^ A similarity measurements, such as pixel-wise difference, correlation, and information theory-based indexes, can also be used to evaluate the difference between the reference frame and the moving frame. An extra regulation term of deformation was often included, to ensure the sparsity of the deformation field^63^ and avoid overfitting. Finally, various optimizers, mostly based on gradient descend theory, were designed to search for the best transformation parameter of the mapping function [Fig. 2(c)]. A thorough review of inter-frame motion artifact correction was given by Oliveira and Tavares.^55^ Intra-frame motion artifacts were commonly corrected based on the Lucas and Kanade^64^ algorithm [Fig. 2(d)] or a hidden Markov model,^65^^,^^66^ where each pixel (or each line) was considered to be sampled from a translated tissue at an independent time-point.^56^^,^^67^ The Lucas and Kanade algorithm expresses the difference between the registered frame and the template frame as the function of the x-y trajectory. The optimal estimation of trajectory was derived by setting the first-order Tayler expansion of the error function to zero. And the algorithm iteratively updates the displacement in the x-y axis, until it converges to an optimal x-y displacement trace which minimizes the difference to the template. To reduce the computational cost, hierarchical approaches of image registration were proposed, where the imaging video was first decomposed into stable and non-stable sections, and different levels of registrations were assigned to the sectioned images.^68^

Apart from lateral motion, axial drifting eventually drew the researcher’s attention with the evolution of increasing imaging time and resolution demand. In 2D parallel imaging, z axial motion correction could be accomplished by a hardware-implemented strategy of real-time focal plane adjustment,^69^ or by computational approaches of calculating the correlation between the time-sequence z slices and a reference cube.^61^^,^^65^ In high-speed volumetric imaging, the defocus problem is naturally resolved in a certain axial range because it could capture multi-focus planes at high speed. The z axis displacement could be equivalently resolved like the x-y displacement by simply adding one more dimension to the registration pipeline. Since the axial displacement could not be avoided completely, volumetric imaging is much more robust to 3D motion artifacts compared with plane-scanning or point-scanning approaches.

A second image processing issue is denoising. Higher-SNR images are always preferred since it enhances the efficiency and fidelity of neuron detection and signal extraction. The simplest solution to acquire a higher-quality image was to use higher laser intensity. But it comes with photobleaching, nonlinear phototoxicity, and heating damage, which interferes with the fundamental neurophysiological phenomenon.^70^ Meanwhile, it is worth noticing that under lower light conditions, photon shot noise becomes comparable to the readout noise of the camera sensor, where the hardware implementation could no longer eradicate noises. Therefore, to facilitate subsequent analysis, data-driven methods become critical.

Image denoising is routinely done by balancing the data fidelity and prior knowledge.^71^ Commonly used priors in fluorescence imaging include sparsity prior,^72^ also known as low-rank prior,^73^^,^^74^ and sensor physics-based noise distribution prior.^75^ Sparsity prior implies that spatial-temporal adjacent blocks in the image would have similar distributions. In other words, high-frequency components are dominated by unwanted noises and should be suppressed selectively. Different methods were used to exploit the sparsity priors. The most general method is to add a regularization term to the image reconstruction target function, which constrains the coefficient density in the transformation domain.^76^ The deconvolution or image reconstruction optimization problem subsequently becomes a multi-target problem. The multiple targets could be decoupled under an alternating direction method of multipliers framework, leading to hybrid iterations of image reconstruction and denoising,^73^^,^^77^ where the data fidelity term and the sparsity term are optimized iteratively. Within the sparsity sub-step, the parameters of the data fidelity term were fixed and the sparsity prior term was optimized, vice versa in the fidelity sub-step. There are various strategies to meet the sparsity prior. A straightforward approach is setting a threshold (soft or hard) cut-off in transformed domain^72^^,^^75^^,^^78^^,^^79^ (Fourier transformation domain, cosine transformation domain, wavelet transformation domain, or a learned over-complete dictionary). The cut-off suppresses the high-frequency components and makes sure that the image frequency spectrum dominantly concentrates on low-frequency bands. Sparsity could also be attained using block-matching approaches. In block-matching-based algorithms, spatial-temporal adjacent blocks within the searching window were vectorized and concatenated into a matrix. The singular value decomposition was performed on the matrix, and then a hard or soft threshold was set to suppress the low-energy components.^80^ It was proved that this kind of strategy could efficiently reduce noise and preserve the details.^72^^,^^78^

Deep-learning methods have facilitated great advances in bioimage denoising.^81^ Artificial neural networks were used to explore the underlying features and recover the noise-degraded images efficiently. There are two kinds of training strategies to train a network, supervised and unsupervised. The supervised strategy requires corresponding noisy and noise-free images to work as training data, and iteratively optimizes the network parameter using gradient descent strategy. In structural imaging *in vivo*, the training dataset could be acquired by pre-tests, where laser-insensitive specimens are used as subject.^82^ And the pre-trained network could then be used to denoise the laser-sensitive specimen under low light conditions. While in functional imaging, this strategy might be problematic due to the non-repetitiveness of calcium transients, leading to the lack of training data for supervised learning. Therefore, self-supervised training was applied in functional neural image denoising issues. The network used solely noisy data as input and output in training. By utilizing the Noise2Noise framework^83^ and temporal redundancy prior,^84^ the network successfully produced noise-free images in test sessions. Thus, the self-supervised framework overcomes the obstacle of dataset shortage and is also practical in functional neural image missions.

Resolution maintenance is a vital concern in denoising algorithms designed for microscopy. Overall, the state of art frameworks such as local block-matching strategy and deep-learning network have less resolution loss compared with conventional transformational domain cut-off algorithms. But the performance is usually sample-specific and optical system-specific, and could not be easily summarized. The robustness is particularly a vital problem in deep-learning methods. Actually, there is a risk of resolution loss in every denoising algorithm because imaging denoising is essentially an ill-posed inverse problem. And there is a fundamental trade-off between detail preservation and denoising performance, in other words, the data fidelity and the prior knowledge. The ability of resolution preservation depends mainly on the accuracy of modeling the signal distribution and noise distribution, which requires the expertise on imaging system and sample property from researchers.

The following step of image preprocess is hemodynamic correction, which is typical in wide-field microscopy but is insignificant in two-photon microscopy. The blood flow in vessels has an impact on the background fluctuation of the neural image, which is exhibited as a large-variance and low-frequency background signal component. This component is calcium-irrelevant and should be removed. The most common approach to correct the hemodynamic is adding an extra reference channel with a specific excitation wavelength whose emission is calcium-independent.^16^^,^^85^ The captured fluorescence intensity was divided by the reference channel, $F_{c}=\frac{F}{R}$, where $F_{c}$ is the corrected signal; F is the calcium channel signal and R is the reference channel signal. The corrected normalized signal could be further expressed as $\Delta F_{c}=\frac{Fc-F_{c0}}{F_{c0}}=\frac{F/R}{F_{0}/R_{0}}-1=\frac{\frac{F_{0}(1+\Delta F)}{R_{0}(1+\Delta R)}}{\frac{F_{0}}{R_{0}}}-1=\frac{1+\Delta F}{1+\Delta R}-1\approx \Delta F-\Delta R$, where subscript 0 stands for averaged signal; $\Delta F=\frac{F-F_{0}}{F_{0}}$, $\Delta R=\frac{R-R_{0}}{R_{0}}$; the last equation is derived through a first order Taylor expansion. Another approach without extra reference channel is modeling the hemodynamic as background signal while performing calcium extraction. This computational approach relies on priors of calcium trace patterns and has no extra hardware cost. Detailed descriptions can be found in the next section.

### 2.2 Neuron Segmentation and Calcium Trace Extraction

After the image processing pipeline, the fluorescence images are low-noise and spatially settled and are ready for neuron segmentation and signal extraction. This session describes the methods used to extract fluorescence traces (or equivalent spike trains) and spatial footprints from the raw optical images. The data size should thus be reduced to several MBs which filters out the background fluorescence from neuropils, gliocytes, hemodynamic, and out-of-focus background while keeping all the neural-coding information intact. Compared with the raw optical video which might be up to hundreds of GBs or even TBs, this process could be regarded as data compression or dimensionality reduction. Further neural functional and network inference could entirely rely on the extracted spatial-temporal footprint matrix.

An intuitive way of signal extraction is first segmenting individual neurons as regions of interest (ROI), and then calculating the (weighted) average temporal brightness of these pixels as the temporal trace. This kind of approach relies heavily on the imaging quality and meticulous identification of the shape and size of the neurons, which could be diverse between different bio-sensors and imaging modalities and often requires the expert knowledge of the researcher. Analytical approaches would assume neuron somas to be roughly circular in shape and flicker at a certain frequency periodically. Based on these assumptions, several image segmentation methods are used. First, the pixel-wise maximum deviation from the average brightness^11^^,^^86^ or the standard deviation (SD) over time is derived to form an active map, from which neuron ROIs could be highlighted. Segmentation methods are subsequently used, ranging from manual approaches^11^^,^^65^ to automatic algorithms. Manual ROI selection may be laborious but assures high ROI quality, which is suitable for small datasets. There are several software implementations available for manual ROI selections with the aid of automatic initializations, such as ImageJ,^87^ SIMA,^66^ and SamuROI.^88^ Automatic algorithms for the segmentation are mainly based on computer vision theories, such as kernel filtering^89^ and graph-cutting theory.^66^ Deep-learning-based methods have also achieved state-of-art cell-segmentation performance,^90^^–^^93^ while the conventional shortcomings of lack of training dataset, computational cost, and algorithm robustness to different imaging modalities and SNR levels are gradually increasing. Separation of overlapped neurons is a major challenge currently faced by deep-learning approaches. The anisotropic resolution further aggravated this problem. Different deep-learning methods adapted diverse strategies to tackle this issue. For instance, in U-Net^91^ framework, the boundary between cells was artificially inserted into the mask of the training dataset, and the corresponding weight of the ridge was increased to force the network to learn the boundary, so overlapped cells were forced to be split into two non-overlap parts. In STNeuroNet,^92^ overlapped neurons were split using watershed algorithm, and the temporal trace was demixed using a linear regression approach.^94^ This would allow overlapping neurons to be separated spatially and temporally. And in Shallow U-Net Neuron Segmentation (SUNs),^95^ segmentation was done frame-by-frame and followed by a merging procedure, in which case overlapped neurons firing at distinct frames could be separated. In the meantime, recent methods with multi-view imaging and reconstruction^96^ can alleviate the anisotropic resolution, which may also increase the fidelity of segmentations.

**Fig. 3:**
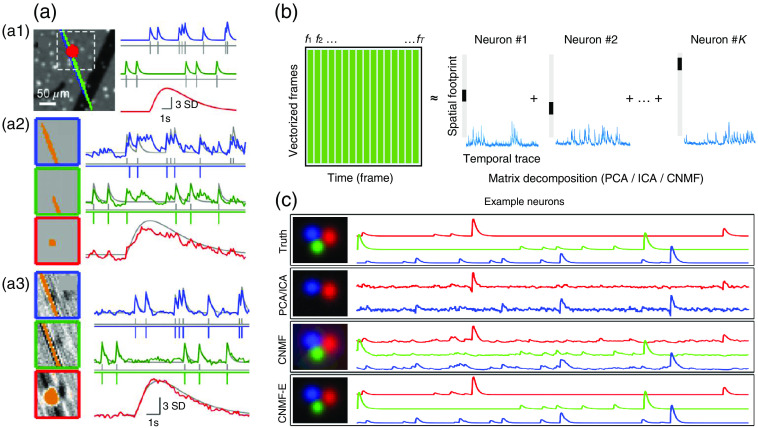
Calcium extraction via ROI-based approaches and matrix decomposition. (a) Comparison of ROI analysis and decomposition methods. The ground-truth responses are marked by gray lines. (a1) A simulated image which contains three spatially overlapping signal sources colored in red (Bergmann glial fibers), blue and green (Purkinje cell dendritic trees). The background contains black vessels and bright interneuron somata. (a2) ROI analysis identified spatial filters (left) of cellular components and their temporal traces (right). (a3) Matrix decomposition method reveals the spatial footprint (left) and their temporal traces (right). The signal estimation is more accurate than the ROI based-approach shown in (a2). (b) Schematic illustration of matrix decomposition methods on neural video. The video matrix was factorized into K rank-one matrixes, each of which stands for the location and temporal activity of an individual neuron. (c) The performance comparisons of PCA/ICA, CNMF, and CNMFE on simulated data. Panel (a) is adapted from Ref. 97. Panel (c) is adapted from Ref. 98.

The main drawback of the active-map-based segmentation is its high missing rate for inactive neurons and neurons with lower fluorescence indicator expression [Fig. 3(a)]. Another commonly used neural detection approach is matrix factorization. The core framework of these methods is to factorize the spatial-temporal matrix $F\in R^{N\times T}$ into spatial component $S\in R^{N\times K}$ and temporal component $A\in R^{N\times T}$ [Fig. 3(b)]. Each column of F containing N pixels is one of the T vectorized frames of the microscopic video. Ideally, S should contain K neurons’ shapes and locations in every column. And A contains the corresponding temporal traces of these neurons. Additionally, there are also noise E and background signal B in F, which could be written as

F=SA+B+E.

If assuming the spatial location of each neuron does not shift through time, which is facilitated by the previous motion registration process, SA could be further factorized into sum of K neuron’s temporal traces. Thus SA could be represented as the sum of K rank-1 matrixes.

$$SA=\overset{K}{\underset{k=1}{\sum }}s_{k}a_{k}^{T},$$

where $s_{k}$ is the k’th column of S, and $a_{k}$ is the k’th row of A.

There are infinite solutions to the factorization problem if non-constrains are set. If using only the least square of error as the criterion, the formula leads us to rank-k approximation problem, which is well solved by singular value decomposition, also known as principal component analysis (PCA). However, the PCA method alone might be ill-suited to extract single-neuron signals, since every principal component might contain information from multiple cells.^97^ A further assumption is made about the statistical independence of each component, which leads to independent component analysis (ICA). ICA was proved to outperform PCA in identifying single-neuron cells.^97^ Sparsity prior is another prior apart from independence. Sparse heterarchical matrix factorizaion^99^ with dictionary learning is used to segment neurons and then cluster them into hierarchical functional clusters and reveal the network structure of functional circuits. Moreover, stricter priors of the shape and calcium response pattern could be made^100^ which narrows down the solution space furthermore. Non-negative matrix factorization (NMF) method puts non-negative constraints on spatial and temporal matrix,^30^^,^^101^ which is intuitive considering the non-negative nature of optical images and calcium activity traces. The NMF method performs better on noisy data compared with ICA and was widely adopted by a series of improved algorithms, such as constrained nonnegative matrix factorization (CNMF),^102^ CNMF for microendoscope data (CNMF-E),^98^ and CNMF with M-estimator to background^103^ [Fig. 3(c)]. However, CNMF with 3D long-term mesoscale video dataset faces the burden of large computational costs up to thousands of GPU-hours.^104^ Several accelerated algorithms are reported, such as seeded iterative demixing (SID)^104^ and online deconvolution of calcium image.^105^^,^^106^ Deep-learning algorithms can also be exploited in the future to further reduce the computational costs with more data priors.^31^

The fluorescence trace normally provides sufficient information as spike trains, since the two modalities are convertible through convolution and deconvolution. However, when precise timing information is wanted, deconvolution may be conducted to improve the data quality.^54^ Under CNMF and OASIS framework, the binary spike train is embedded under the autoregressive model of calcium impulse response and could be achieved through primal optimization. If the segmentation was done using ROI strategies, where spikes were not directly accessible, greedy template fitting was commonly used for spike event detection.^8^^,^^107^^,^^108^

Another common operation on the fluorescence trace is signal normalization, which extracts the mean activity of each neuron and calculates the difference from average score as ΔF/F. These normalizations could further exclude the influence of incoherent background fluorescence and facilitate the analysis of neural functions.

## 3 Functional Mapping and Network Inference

At this phase of analysis, the information from fluorescence video is compressed into a 2D matrix $X\in R^{N\times T}$ containing the temporal traces of each neuron and the spatial maps of their locations on the brain atlas. The i’th row of X represents the temporal trace of the ith neuron, lasting T frames in total. The information encoded in X is, to a large extent, similar to electrophysiological signals. The only difference is that optical signals reflect calcium transient intensity for calcium indicators (or other transient intensity for specific indicators such as voltage and other neurotransmitters), while electrophysiology signals reflect spike trains. These two modalities could be equivalently converted via event detection (deconvolution) and kernel convolution algorithm. Hence, the analysis methods used for these two modalities are generally consistent. In the following sections, we will introduce generic methods on functional mapping and network inference which would be appliable in both electrophysiological and fluorescence signal analysis, unless otherwise noted.

### 3.1 Functional Mapping on Single-Neuron Resolution

An emerging challenge for mesoscale optical recording of neural signals is that task-relevant neurons only take up a small proportion of captured neurons and might be spatially dispersed over the FOV.^109^ Most of the neurons over the cortex encode spontaneous and uninstructed movements unrelated to the task.^110^ Including all these neurons in analyses would cost large computational costs and introduce undesirable noises, which reduce the SNR of the neural encoding space. Thus, a first and foremost step before functional analysis is to discriminate the task-relevant neurons from the massive number of entire neurons.

**Fig. 4:**
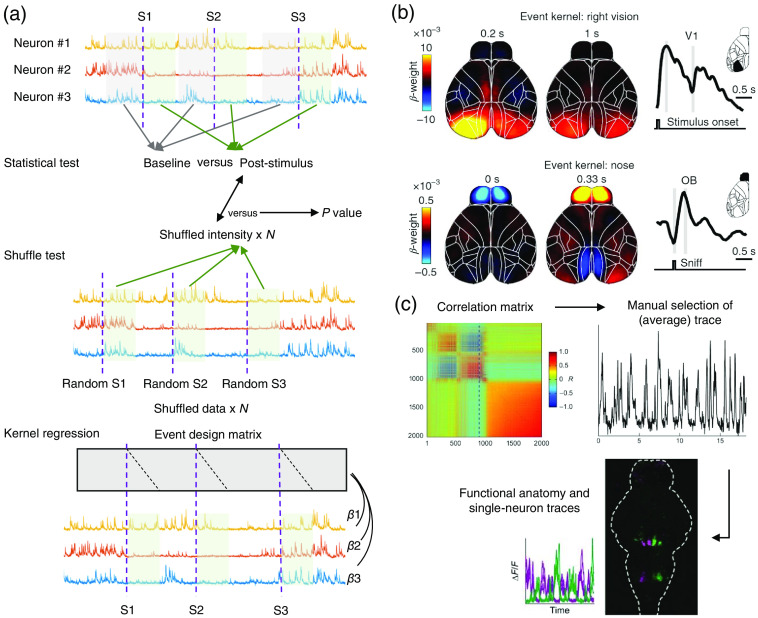
Examples of functional mapping and functional circuit identification. (a) Schematic of task-relevant neuron evaluation. About three example neuron traces are shown with three stimuli as event triggers (dashed lines). In statistical test approaches (top), the fluorescence intensity in the pre-stimulus baseline window (shaded in gray) is compared with post-stimulus time windows (shaded in green), deriving the significance index. In shuffle test approaches (middle), post-stimulus reactions in the shuffled dataset are compared with observed true reaction, where a P-value is defined as the percentile of exceeded shuffled data by the observed data. In the kernel regression (bottom) method, the linear model was used to predict the neuron response from the event design matrix. The link between the task and the neuron activity is evaluated via the regression coefficient β. (b) Example weight maps of event kernel for right visual stimulus and nose movement at pixel-level. (c) Diagram of correlation-based functional clustering at the voxel level. The correlation matrix of the pre-selected supervoxels is manually inspected and a reference trace is selected. Each voxel in the brain is correlated to the reference trace, forming a correlation map. Green, positive correlations; magenta, negative correlations. Panel (b) is adapted from Ref. 110. Panel (c) is adapted from Ref. 60.

A forthright intuition is to select neurons that exhibited a more active firing pattern during the task [Fig. 4(a)]. The degree to which task events modulate neuron activities could be evaluated through statistical significance tests by comparing the averaged firing rate between task trials and baseline.^109^ Diverse time windows could be used, such as pre-stimulus, post-stimulus, and post-movement onset, depending on the interested task variable. However, the significance test employs nothing on the unique distribution of individual neuron activity prior. Neurons with periodic firing or with extremely active or inactive firing properties may be misjudged by statistical tests. Thus, an alternative option is to perform a shuffle test on the same neuron’s activity trace. Shuffling the start time stamp of behavior segments^11^ or shuffling the timepoint of calcium events^8^ are both feasible. The same firing rate average analysis is performed on the shuffled data. The significance of task modulation is evaluated through how many percentiles of the shuffled data the primary data could exceed.

Significance tests revealed the overall task modulation on the firing rate during a task period. But within a complicated task, there might be more than one task variable, such as engagement, choice, precision, and body gestures. Statistical tasks based on mean firing rate perform badly on separating these fine variables or revealing the non-linear neural representation. To uncover the relationship between single-neuron activity and animal behaviors, superior data analysis methods should be employed.

Linear regression, also known as general linear modal is an effective method for exploring data correlations. The regression modal could be expressed as y=Xβ,^108^ where β is a concatenated event kernel vector to be regressed. $X\in R^{T\times L}$ is a Toeplitz design matrix concatenated by K events of interest $X_{k}$, where $X_{k}\in R^{T\times L_{k}}$, $\overset{K}{\underset{k=1}{\sum }}L_{k}=L$, $X=[X_{1},X_{2},\ldots ,X_{K}]$. $X_{k}$ contains diagonal 1s at each event onset and corresponding time lags and 0 elsewhere. y is the normalized neuron trace time course as the prediction output. The link strength between the neuron trace and the behavior variable could be evaluated through the coefficient in β. A larger absolute value in β stands for a higher correlation between neuron and behavior. The spatial-temporal map of β could also indicate the information flow between different neuron clusters. To avoid overfitting, different regulation terms are added to rescript the sparsity of the coefficient, which make the model more practical, such as reduced-rank kernel regression,^109^ ridge regression^108^ or lasso regression^110^ [Fig. 4(b)].

In perceptual pathways, such as tactile, olfaction, and vision, neural coding mechanisms are often better explored, thus allowing more accurate and complicated modeling of neural activities. For instance, in the tactile sensory pathway, the functional mapping modal assumes that the sensory input goes past static, point-wise nonlinearity, and is convolved with a temporal kernel before being input to a Gaussian noise model.^8^ While in the olfaction pathway, different olfactory is believed to be encoded by population vector instead of single-neuron activities.^9^ And in the visual pathway, the ganglion neurons are well known to be modulated by surrounding neurons, forming the widely recognized receptive field.^111^ These models successfully predict the activity in the local circuit with high accuracy and specificity.

Comparing the modal-free analysis with the model-based analysis, the former is more helpful for rough neuron sorting from mesoscale images, while the latter uncovers the explainable mechanism of neural encoding. Thus, we advocate taking advantage of the high data-throughput property of mesoscale imaging as a neural information database, from which neuron clusters could be distinguished and separated depending on their functions. While the subsequent modeling of neural encoding could be more targeted and prior-based. This may extend the boundary of controversial single-brain-area functional mapping restriction.

### 3.2 Brain-Wide Single-Neural Level Functional Network Study

It is widely recognized that different levels of clusters of neurons are recruited for diverse behavior tasks.^112^ Even a simple perceptual input could arouse distributed neural activities across the brain. Communication across multiple regions is crucial for the brain functioning as a system.^29^^,^^109^^,^^113^ Observing the brain *in vivo* with behaving animals enables the inference of functional circuits across brain areas, by analyzing statistical dependencies between neurons.

Restricted by the detection capability, conventional network inference either concentrated on a local circuit or utilized low-spatial-resolution brain activities through functional magnetic resonance imaging,^113^^,^^114^ electroencephalogram, or wide-field microscopy.^29^ Brain activities acquired through these techniques characterize the area-averaged multi-unit neuron activity containing multi-frequency oscillation components,^115^ thus are temporally smoother and more stable across trials. Single-neuron activities, however, exhibited larger variance, sparsity, and randomicity through trials.^116^ The commonly used spectrum methods on field potentials decompose different frequency components and analyze the phase lag between brain areas to infer the information flow through the precedence of brain areas. These methods became meaningless on single-neuron traces because the frequency components lack interpretable physical meanings. Metrics of similarity such as instantaneous phase, phase lag, and phase synchronization index^117^ are no longer applicable. And the quadratic increase of the computational complexity while calculating pair-wise similarity of the neurons also brings challenges to the network inference. All these barriers bring great difficulties and opportunities to interpret the working mechanisms of neural circuits at the single-neuron level with mesoscale neural imaging.

Despite the inefficiency of phase decomposition-based methods, the covariance or correlation remains the most straightforward model-based metric which describes the undirected similarity between two fluorescence signals. The similarity matrix represents the pairwise correlation among the neural population. With the pairwise correlation which describes the similarity of neurons, subsequent clustering^118^ methods could be used to assemble the neurons into separated groups.^119^ Manually selected seed with Pearson correlation matrix has been used to identify the functional circuits across the larval zebrafish brain^60^ [Fig. 4(c)]. There are plentiful other unsupervised clustering methods available, such as k-means clustering, hierarchical clustering, barcode analysis, and graph-based analysis. These methods have been used in different experiments. The k-means clustering was used to find the representation structure at single-neuron level in the rat orbitofrontal cortex.^120^ The k-means method remains computationally efficient even with high dimensional data, but the result of k-means algorithms depends heavily on the hyperparameter k, thus requiring pretest or the prior knowledge from the researcher. The hierarchical algorithm was used to cluster the retinal ganglion cells and exhibited high interpretability and visualization ability,^121^ However, it may encounter severe computational complexity with higher-dimensional data. The barcode analysis required a character barcoding process before categorizing. For instance, the neurons from the whole brain of the zebrafish were barcoded via their response to certain stimuli and clustered into 256 classes.^86^ What may be troublesome with barcoding is that the number of categories increases exponentially with the bit of barcode in binary coding, which restricts its application scope.

Apart from the model-based clustering, functional cell assemblies could also be identified through model-free decomposition analysis. Factorization methods are performed on the 3D tensor stacked by multi-trial 2D neural activity maps, which includes PCA, demixed principal component analysis (demixed PCA),^122^ and non-negative tensor factorization.^123^ These models factorize the tensor into rank-1 components and extract the temporal and trial dimensional vector as trial-consistent and within-trial latent variables of the neuron representation. Though unsupervised, these vectors may correspond to behavioral observations such as performance accuracy and task engagement.

The temporal correlation-based network mentioned above often induces false-positive connections between indirectly coupled neurons.^112^ A sparser binary adjacency matrix could be derived by hard-threshold or k-nearest neighbors. On the other hand, Granger causality, or transfer entropy analysis, is a temporal-precedency-based metric that reveals directed information flow from one node to another. Its basic assumption is that the history of a precedent neuron should be contributing to predicting the future of the downstream neuron. Granger causality has been used to investigate the unbalanced distribution of information density in the somatosensory cortex of the mouse.^124^ Additionally, there has been open-source algorithm toolboxes^125^ for performing Granger causality analysis. The computational cost of Granger causality algorithm increases quadratically with the number of nodes. Improved Granger causality algorithms^126^ are developed to decrease computational costs and avoid overfitting. Transfer entropy also serves as an equivalent for Granger causality under Gaussian variables^127^ with a much lower computational complexity. With the help of optogenetics,^128^ it is possible to perturbate *in vivo* neurons and watch the network in dynamic. Modal-based Bayesian network was used to inference the neural spike trains and connectivity with simultaneous optogenetic perturb and electrophysiological recording.^129^

## 4 Looking Ahead

Mesoscale imaging with single-neuron level resolution has provided unprecedented potential for understanding the mechanism of both local and long-range brain circuits. Ambitious computational scientists look forward to mimicking the brain functions and building general intelligent machines. But there are still data analysis obstacles lying ahead of us, which would take long-term efforts before we uncover the myths of the brain network.

The fast-growing of long-term high-speed mesoscale volumetric imaging craves high-efficiency data analysis methods. The volume sequences captured by light-sheet microscopes, light-field microscopes, or multifocal microscopes could easily make TB-level data sizes. For a typical example, conventional matrix decomposition approaches are ineffectual for calcium extraction from 3D volumes. SID^104^ was used to reconstruct 3D positions of neurons by first deconvolving the pixel-wise SD image of background-subtracted images. The neuron candidate positions were identified using a band-pass filter and back-projected to multiple views for spatial-temporal footprint update. This has enabled the decomposition to take place in a lower dimension, which saves vastly the computational cost. Further analysis considering the pairwise correlation or causality between neurons also costs massive computing resources. Apart from the task-relevant sorting procedure, which could massively reduce the analyzing subjects, dimensionality reduction under sparsity prior^118^ could also help us with identifying hierarchical clusters of neuron assemblies. Nevertheless, analyzing the populational property of neurons increases the stability over trial and time-lapse, and surrenders the single-neuron level calculation mechanism, which should be a trade-off that requires careful consideration. Data-driven deep-learning methods are very promising in many steps of the whole pipeline especially for its low computational costs, since the mesoscale imaging data usually has very strong local similarity across a large FOV. The whole pipeline should be considered simultaneously during the design of the network framework, while the downstream analysis can also be used as the metric to evaluate the performance of different algorithms. The generalization and practicality of current deep neural networks need to be enhanced for its broad and convenient applications. In addition, more and more mesoscale databases of different imaging modalities and diverse tasks are required to promote this field and evaluate the rapidly emerging algorithms.

Another concern of single-neuron network study, as well as higher-level networks inference of brains, is the network interpretability.^130^ There are researchers arguing the credibility of the commonly used methods such as single-neuron lesion, correlation, and granger causality.^131^ The analytic approaches were performed on a microprocessor whose algorithm flowchart was known as prior. The research concluded pessimistically that current approaches may fall short of interpreting the algorithm of the neural system, regardless of the amount of data. It seems that although we are desperate for acquiring more data, the basic tool of understanding the brain network is still absent. It was suggested by Marr^132^ that there are three levels of understanding a system: functions, algorithms, and implementation. Although we have dissected several perceptual input pathways such as vision and audition, the basic communication protocol of neurons remained unknown. To understand how the neurons in brain give rise to the ability of recognition, we may need a network analysis technique that could find more fundamental arithmetic units or their hierarchical structures of neural network.

In conclusion, we have introduced the general analysis pipeline of fluorescence mesoscale brain images in this review. But there are still multiple challenges for existing methods to deal with the complex network of the brain. Nevertheless, we believe that with the rapid growth of imaging techniques, analyzing methods, and computational abilities, global circuits on the single-neuron level will be more extensively explored in the future.

## Code, Data and Materials Availability

Example data and code in this paper are currently not publicly available, but may be obtained from the author upon reasonable request.
